# WIP1 Phosphatase as a Potential Therapeutic Target in Neuroblastoma

**DOI:** 10.1371/journal.pone.0115635

**Published:** 2015-02-06

**Authors:** Mark Richter, Tajhal Dayaram, Aidan G. Gilmartin, Gopinath Ganji, Sandhya Kiran Pemmasani, Harjeet Van Der Key, Jason M. Shohet, Lawrence A. Donehower, Rakesh Kumar

**Affiliations:** 1 Oncology R&D, GlaxoSmithKline, 1250 S. Collegeville Road, Collegeville, Pennsylvania, United States of America; 2 Department of Molecular Virology and Microbiology and Department of Pediatrics, Baylor College of Medicine, Houston, Texas, United States of America; 3 Ocimum Biosolutions Ltd., HUDA Techno Enclave, Hyderabad, AP, India; 4 Platform Technology & Science, GlaxoSmithKline, 1250 S. Collegeville Road, Collegeville, Pennsylvania, United States of America; 5 Texas Children’s Cancer Center, Department of Pediatrics, Baylor College of Medicine, Houston, Texas, United States of America; Centro Cardiologico Monzino, ITALY

## Abstract

The wild-type p53-induced phosphatase 1 (WIP1) is a serine/threonine phosphatase that negatively regulates multiple proteins involved in DNA damage response including p53, CHK2, Histone H2AX, and ATM, and it has been shown to be overexpressed or amplified in human cancers including breast and ovarian cancers. We examined WIP1 mRNA levels across multiple tumor types and found the highest levels in breast cancer, leukemia, medulloblastoma and neuroblastoma. Neuroblastoma is an exclusively *TP53* wild type tumor at diagnosis and inhibition of p53 is required for tumorigenesis. Neuroblastomas in particular have previously been shown to have 17q amplification, harboring the WIP1 (*PPM1D*) gene and associated with poor clinical outcome. We therefore sought to determine whether inhibiting WIP1 with a selective antagonist, GSK2830371, can attenuate neuroblastoma cell growth through reactivation of p53 mediated tumor suppression. Neuroblastoma cell lines with wild-type *TP53* alleles were highly sensitive to GSK2830371 treatment, while cell lines with mutant *TP53* were resistant to GSK2830371. The majority of tested neuroblastoma cell lines with copy number gains of the *PPM1D* locus were also *TP53* wild-type and sensitive to GSK2830371A; in contrast cell lines with no copy gain of *PPM1D* were mixed in their sensitivity to WIP1 inhibition, with the primary determinant being *TP53* mutational status. Since WIP1 is involved in the cellular response to DNA damage and drugs used in neuroblastoma treatment induce apoptosis through DNA damage, we sought to determine whether GSK2830371 could act synergistically with standard of care chemotherapeutics. Treatment of wild-type *TP53* neuroblastoma cell lines with both GSK2830371 and either doxorubicin or carboplatin resulted in enhanced cell death, mediated through caspase 3/7 induction, as compared to either agent alone. Our data suggests that WIP1 inhibition represents a novel therapeutic approach to neuroblastoma that could be integrated with current chemotherapeutic approaches.

## Introduction

The p53 pathway plays a critical role in maintaining the genomic fidelity of stem cell populations, including neural stem/ progenitor cells [1,2]. Wild-type p53-induced phosphatase 1 (WIP1/ PPM1D) is a type 2C serine/ threonine phosphatase that negatively regulates key DNA damage response proteins such as p53, ATM, and p38MAPK [3]. Loss of WIP1 in mice causes deficiencies in various self-renewing cell populations resulting in defects including T cell maturation and male fertility [4–6]. Conversely, conditional overexpression of WIP1 in the brains of aging mice can promote sustained elevated neurogenesis [7]. WIP1 expression has been demonstrated in neural stem cells, and higher levels of WIP1 and reduced p53 activation are associated with increased neurogenesis during maturational stages of growth [8].

Genomic instability stemming from dysregulation of cell cycle checkpoints and the DNA damage response is a common theme in cellular transformation. Late stage tumors often show loss of phosphorylation of DNA damage responsive proteins, suggesting that inactivation of this pathway is a prerequisite for cancer progression [9,10]. WIP1 has been shown to play a role in the homeostatic downregulation of resolved DNA damage responses in various healthy tissues, and also acts as an oncogenic inhibitor of multiple tumor suppressors during cancer progression, highlighting the importance of WIP1 in maintaining genome stability. Following DNA damage, p53 becomes activated and subsequently upregulates WIP1 which inactivates, via dephosphorylation, multiple effectors of the DNA damage response, including p53 (reviewed in [11,12]). Functionally, WIP1 (*PPM1D*) behaves as an oncogene, contributing to the transformation by other driver oncogenes in *in vitro* transformation assays and in genetically engineered mice [4,13,14]. The importance of WIP1 in human cancers is evident from the fact that it is amplified and overexpressed in primary breast tumors [15–17], gastric carcinomas [18], medulloblastoma [19–21], neuroblastoma [22], ovarian clear cell adenocarcinomas [23,24] and pancreatic adenocarcinomas [25]. [25]. Amplification of the 17q locus containing *PPM1D*/ WIP1 and gain of function mutations occur in multiple pediatric tumors including gliomas [26], medulloblastomas [19], and neuroblastomas [22]. Activating germline mutations in the *PPM1D* gene have recently been associated with predisposition to breast and ovarian cancer [27]. These carboxy terminal truncating mutations have also been identified in pediatric gliomas and colorectal tumors [26,28].

Here, we focus on the role of PPM1D in neuroblastoma pathogenesis. This gene is amplified in a small subset of tumors and over expression of WIP1 has been shown to correlate with poor outcome [22]. Importantly, the vast majority of neuroblastoma tumors are p53 wild-type at diagnosis, suggesting that repression of p53 function is critical for tumorigenesis [29]. In addition, repression of p53 functions by high levels of MYCN and via MDM2 has been shown to be a critical step in MYCN driven malignant transformation of neuroblastoma precursors [30]. Thus, there is a clear rationale for de-repression of p53 function as a therapeutic approach to this highly aggressive pediatric cancer [31,32].

We have recently reported the identification and characterization of a novel series of allosteric, small molecule WIP1 antagonists including the orally bioavailable compound, GSK2830371 [33]. Here, we report that inhibition of WIP1 selectively impairs the growth of *TP53* wild-type neuroblastoma cell lines. In addition, WIP1 inhibition markedly sensitizes *TP53* wild-type neuroblastoma cell lines to genotoxic chemotherapy. These data suggest that WIP1 meditated repression of p53 is a novel therapeutic target for neuroblastoma.

## Materials and Methods

### Gene expression data analysis

Quantile-normalized values from Human Genome U133 GeneChip arrays (Affymetrix, Santa Clara, CA) corresponding to *PPM1D* probeset (204566_at) were analyzed using Oncomine Powertools (Oncomine v4, Life Technologies, Ann Arbor, MI) across >25K samples. Overexpression was determined by setting the median to 1 and filtering values 4 fold above the median per tumor type.

### PPMID expression in Neuroblastoma Cohorts

Analysis of PPMID was performed using the R2: microarray analysis and visualization platform (http://r2.amc.nl). The Neuroblastoma public-Versteeg-88 patient cohort [34] consists of 88 patients with annotated survival and tumor stage information as well as gene expression data.

### *In vitro* Phosphatase Assay

p53 pS15 (Ac-VEPPLpSQETFS-amide), p38 MAPK Thr180 (Ac-TDDEMpTGpYVAT-Amide), CHK1 pS345 (Ac-QGISFpSQPTCP-amide), CHK2 pT68 (Ac-LETVSpTQELYS-amide) and H2AX pS139 (Ac-GKKATQApSQEY-amide) phosphopeptides used were synthesized by New England Peptide (Gardner, MA). The RRA(pT)VA phosphopeptide is a component of the Serine/Threonine Phosphatase Assay System (Promega, Madison, WI). One hundred ng of each phosphatase was diluted in PP2C buffer (50 mM Tris-HCl pH 7.5, 0.1 mM EGTA and 0.02% 2-mercaptoethanol) and incubated with 1 mg/mL BSA, 30 mM MgCl2 and 100 μM of the specified phosphopeptide for 1 hour at room temperature. Free phosphate released from the reaction was determined using BIOMOL Green reagent (Enzo Life Sciences, Farmingdale, NY) according to the manufacturer’s protocol. Optical density at 630 nm was detected on a Victor2 multilabel reader (Perkin Elmer, Waltham, MA). Recombinant WIP1 was purified as previously described using the WIP1-ΔExon6-His-pET-23a+ construct kindly provided by Dr. Ettore Appella [35]. To obtain p53 proteins for *in vitro* phosphatase assays on full-length p53 proteins, HEK293 cells were transfected with a flag-tagged p53 expression plasmid (Addgene plasmid #10838 supplied by Dr. Thomas Roberts) and treated with 10 Gy ionizing radiation followed by a 30 minute recovery. Flag-tagged p53 proteins were subsequently immunopurified using anti-flag antibody conjugated beads (Sigma Aldrich) and the protein bound to the beads was used as a substrate in the *in vitro* phosphatase assay. The phosphatase activity towards the intact proteins was determined by western blotting using antibodies against p53 phosphorylated at serine 15 (Cell Signaling) and total p53 (Santa Cruz Biotechnology). GST-tagged PPM1A and PPM1B recombinant protein was provided by Dr. Wendell Yarbrough (Yale University). Purified PP2A was purchased from Millipore (Billerica, MA). Purified PPM1G and PPM1H were purchased from OriGene Technologies (Rockville, MD).

### Cell Culture and Compounds

Cell lines tested at GlaxoSmithKline were obtained from the American Type Culture Collection, Manassas, VA (ATCC: IMR-32, SK-N-SH, CHP-212, KAN-TS, SK-N-AS, SK-N-BE(2), and SK-N-FI), Deutsche Sammlung von Mikroorganismen und Zellkulturen, Braunschweig, Germany (DSMZ: SIMA, CHP-134, NBL-S, and SH-SY5Y), and the European Collection of Cell Cultures (ECACC: KELLY and LA1-5S). Cell lines tested at Baylor College of Medicine (Lan-5, NGP and CHLA-255) were supplied by Dr. Metelitsa (Texas Children’s Hospital, Baylor College of Medicine) and validated previously [36]. Neuroblastoma cell lines were grown in RPMI-1640 and supplemented with 10% gamma irradiated and heat inactivated fetal bovine serum (Sigma Aldrich, St. Louis, MO), 1X GlutaMAX (Life Technologies, Grand Island, NY), 1 mM sodium pyruvate (Life Technologies), and 1% Penicillin-Streptomycin (Life Technologies). All incubations were carried out at 37°C, 5% CO_2_, in a humidified incubator. GSK2830371 was synthesized at GlaxoSmithKline [33]. Additional compounds were purchased from external vendors as follows: carboplatin (Sigma Aldrich), doxorubicin (Enzo Life Sciences). All compounds were prepared at 10 mM in DMSO and stored at room temperature.

### RNA Expression Analysis

Cells were exposed to 10 Gy ionizing radiation and harvested at six hours post treatment. RNA was purified using an RNeasy kit (Qiagen Sciences, Germantown, MD). One μg of RNA was used for cDNA synthesis using qScript cDNA Supermix (Quanta BioSciences, Gaithersburg, MD) according to the manufacturer’s protocols. One tenth of the cDNA reaction was used for quantitative RT-PCR using SYBR Green PCR Mastermix (Life Technologies) according to the manufacturer’s protocol in a Step One Plus PCR machine (Life Technologies). Sequences for primers used are: GAPDH forward 5’-ggagtccctgccacactcag-3’ and reverse 5’-ggcccctcccctcttca-3’, *PPM1D* forward 5’-tagtggtgctcagcctgcaa-3’ and reverse 5’-tctgcgtcgcatggtgagt-3’, CDKN1A forward 5’-cctcatcccgtgttctccttt-3’ and reverse 5’-gtaccacccagcggacaagt-3’, and MDM2 forward 5’-tccccgtgaaggaaactgg-3’ and reverse 5’-tttcgcgcttggagtcg-3’. Target mRNA was normalized to GAPDH and relative expression compared to undamaged samples calculated by the standard ΔΔCT method.

### Proliferation Assays

Cells were seeded into 96 well plates at 500 cells per well and incubated overnight. Serial dilutions of GSK2830371 were added to cell plates. For combination studies, chemotherapeutic agents were added to cell plates one hour after GSK2830371. Cell viability was determined at days 0 and three (combination) or five and seven (single agent) post-drug addition using the CellTiter-Glo cell viability assay (Promega) or Cell Counting Kit-8 (Dojindo Molecular Technologies, Rockville, MD) according to the manufacturer’s protocol. CellTiter-Glo luminescent signal was detected on an EnVision 2104 multilabel reader (Perkin Elmer) and CCK-8 optical density at 450 nm was detected on a Victor2 multilabel reader (Perkin Elmer). Mutually nonexclusive combination index was calculated at the IC_50_ of single agents and fixed ratio combinations using the equation described by Chou [37].

### Immunoblot Analysis

IMR-32 and SK-N-AS cells were suspended at 1.5 million cells per mL, and dispensed into 6 well tissue culture plates in 2 mL volumes. After an overnight incubation, DMSO or GSK2830371 serial dilutions were transferred to duplicate wells of cell plates and incubated for 6 hours. Cells were then washed with ice-cold PBS without calcium chloride or magnesium chloride, and lysed in ice-cold RIPA lysis buffer (Teknova, Hollister, CA) containing Complete Mini protease inhibitor and PhosSTOP phosphatase inhibitor (Roche Applied Science, Indianapolis, IN). Lysates were briefly sonicated, clarified by centrifugation, and total protein was determined (BCA Protein Assay, Thermo Scientific, Rockford, IL). SDS-PAGE was performed using pre-cast 4–12% Bis-Tris or 3–8% Tris-Acetate gels with the Novex NuPAGE system (Life Technologies). Western transfer to nitrocellulose membranes was carried out using the iBlot dry blotting system (Life Technologies) set to 9 minutes or wet transfer using the Novex NuPAGE system at 30 V (1 hour). Membranes were blocked with Odyssey Blocking Buffer (Li-Cor Bioscience, Lincoln, NE) for one hour prior to being probed with the following antibodies from Cell Signaling Technology (Danvers, MA, USA): p53 (S15), H2AX (S139), H2AX, p21, CHK1 (S345), CHK1, CHK2 (T68), CHK2, ATM (S1981), ATM, p38 (T180/Y182), p38, and PUMA. Membranes were also probed with antibodies to WIP1 (Bethyl Laboratories, Montgomery, TX) and p53 (Millipore). A GAPDH antibody (Abcam, Cambridge, MA) was used to correct for protein loading. Secondary antibodies conjugated to IRDye 680 and 800 (Li-Cor Bioscience) were used for detection. Antibodies were diluted in 50% Odyssey Blocking Buffer, 50% PBS, 0.1% Tween-20. Membranes were probed overnight with primary antibodies and one hour with secondary antibodies. Odyssey molecular weight markers were used to determine protein size (Li-Cor).

### Copy Number Analysis

*PPM1D* (VPH117-0293702A; VPH117-0293389A; VPH117-0293627A) and *MYCN* (VPH102-0080415A; VPH102-0080404A; VPH102-0080430A) copy number status were determined by running the Qiagen qBiomarker Copy Number assays (Qiagen, Valencia, CA). Assays were performed in quadruplicates using 2 ng of genomic DNA per reaction following manufacturer’s protocol (Qiagen) on a QuantStudio real time PCR instrument (Life Tech, Carlsbad, CA). Data was normalized to a multi-copy reference assay (VPH000-0000000A) and analyzed by the calibrator genome method (Qiagen’s software).

### Geneset Enrichment Analysis of Differentially Sensitive Cell Lines

Publicly available Affymetrix microarray data for the cell lines used in this study were downloaded from Cancer Cell Line Encyclopedia (CCLE: http://www.broadinstitute.org/ccle/home) and NCBI's Gene Expression Omnibus (GEO: http://www.ncbi.nlm.nih.gov/geo/) (see Table 1). CEL files were processed by RMA, and log_2_ transformed data was subjected to Gene Set Enrichment Analysis (GSEA) using the tool developed by the Broad Institute (http://www.broadinstitute.org/gsea) [38]. Select *TP53* signatures extracted from MSigDB collection (http://www.broadinstitute.org/gsea/msigdb/) were interrogated by GSEA following geneset permutations in cell lines sensitive or resistant to GSK2830371 (see Table 1) using a signal-to-noise statistic. Statistical significance, enrichment scores and plots were generated for 1000 random permutations.

**Table 1:** Molecular and pharmacological characteristics of cell line panel.

Cell Line	*TP53* Status	IC_50_ (nM)	Sensitive (S) / Resistant (R)^#^	*PPM1D* Copy Number^	*MYCN* Copy Number^
SIMA*	Wild-type	121	S	Gain	Amplified
IMR32*	Wild-type	259	S	Gain	Amplified
SK-N-SH*	Wild-type	322	S	Gain	Gain
CHP-134*	Wild-type	377	S	Gain	Amplified
NBL-S	Wild-type	518	S	Normal	Gain
Lan-5*	Wild-type	858	S	Gain	Amplified
CHP-212*	Wild-type	1,546	S	Gain	Amplified
KanTS	Wild-type	4,723	R	Gain	Amplified
SH-SY5Y*	Wild-type	5,602	R	Gain	Amplified
CHLA-255	Wild-type	6,322	R	Normal	Gain
NGP*	Wild-type	> 10,000	R	Gain	Amplified
Kelly*	Pro177Thr	> 10,000	R	Gain	Amplified
LA1-5s	Cys182STP	> 10,000	R	Normal	Amplified
SK-N-AS*	Del ex9/in10 junction	> 10,000	R	Gain	Normal
SK-N-BE(2)*	Cys135Phe	> 10,000	R	Normal	Amplified
SK-N-FI*	Met246Arg	> 10,000	R	Gain	Gain

SK-N-AS data from Nakamura, et al, *Biochem Biophys Res Commun* 2007, 354:892–898.

*Cell lines used in GSEA analysis – Microarray data for NGP, CHP-134, Lan-5 were obtained from GEO (GSE28019) and the rest were downloaded from CCLE

^#^Sensitivity cut-off is 3 μM (10 X average of lowest five IC_50_ values)

^Normal: 2 copies; Gain: between 2–6 copies; Amplified: >6 copies

## Results

### WIP1 mRNA is Overexpressed in Neuroblastoma and Medulloblastoma

**Fig 1:**
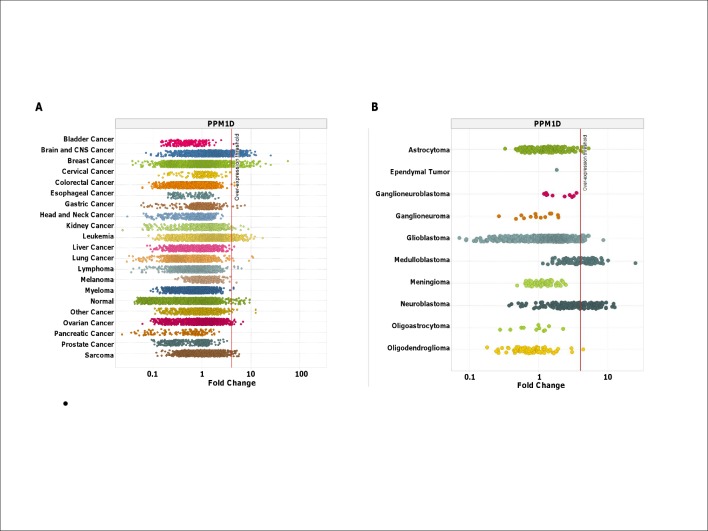
WIP1 is frequently overexpressed in neuroblastomas. **A**, Survey of *PPM1D* (WIP1) mRNA expression from >25K microarray expression profiles across multiple human tumors, compared to composite of all normal tissues (detailed in S1 Fig.). **B**, *PPM1D* (204566_at) is frequently overexpressed in neuroblastoma and medulloblastoma among brain and CNS malignancies. Here, overexpression measured in tumor tissues is determined above the indicated threshold (see Materials and Methods).

**Fig 2:**
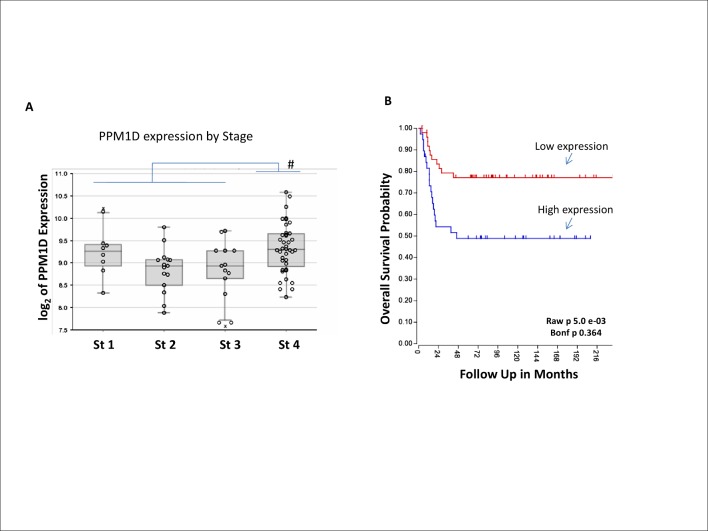
WIP1 expression correlates with neuroblastoma disease stage. **A**, Analysis of a large patient cohort (88 patients) with annotated clinical data and long term follow up (Versteeg-88-Mas5.0) demonstrates higher *PPM1D* levels in stage 4 metastatic subgroup (# = p < 0.01, # # = p < 0.005, by Student T-test). **B**, Long-term survival was also significantly (Kaplan-Meier analysis p = 0.005) different for high (n = 50) versus low (n = 38) expression of *PPM1D*.

During our initial characterization of WIP1 inhibitor’s cellular activity [33], we noted that sensitive cell lines fell within two broad categories: (i) hematological malignancies with a high frequency of wild-type *TP53* but normal *PPM1D* copy number and expression, exemplified by AML, and (ii) those derived from solid tumors with both wild-type *TP53* and aberrantly high WIP1 levels. To expand upon these observations and further define a potentially responsive clinical population, we used publicly available microarray data in Oncomine to determine the level of WIP1 mRNA expression across several different tumor types. Our results confirm that, in addition to breast cancer, nervous system malignancies have a high frequency of elevated WIP1 expression, relative to the cognate normal tissue (Fig. 1A and S1 Fig.). Neuroblastoma and medulloblastoma, in particular, have the highest WIP1 expression within this tumor histology type (Fig. 1B). Further analysis of *PPM1D* gene expression in neuroblastoma using the R2 genomics platform of annotated databases (http://r2.amc.nl) demonstrates a correlation of *PPM1D* mRNA level with higher stage tumors (Fig. 2A) as well as with worse long-term overall survival in a cohort of 88 patients (48% survival versus 78% survival, P < 0.005) (Fig. 2B). These data suggest that WIP1 mediated inactivation of p53 may impact tumor progression to higher stage disease and possibly response to chemotherapy. Consistent with this finding, a number of neuroblastoma cell lines harbor amplifications of the *PPM1D* gene [22]. This is of particular interest since genomic gains in 17q are associated with advanced stage neuroblastoma.

### GSK2830371 is a Selective Inhibitor of WIP1 Phosphatase Activity

**Fig 3:**
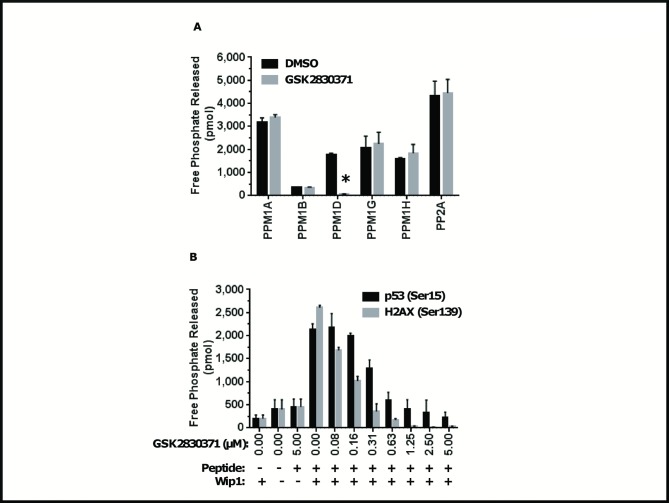
GSK2830371 selectively inhibits WIP1 among related phosphatases. **A**, Release of free phosphate was measured after incubation of each recombinant enzyme with the generic phosphopeptide, RRA(pT)VA. (* p < 0.01). **B**, GSK2830371 inhibits the dephosphorylation of WIP1 substrates. Release of free phosphate was measured after exposure of PPM1D (WIP1) to increasing concentrations of GSK2830371, followed by a reaction with phosphopeptide substrates, H2AX (Ser-139) and p53 (Ser-15). Error bars represent SD.

WIP1 is a member of the PPM family of magnesium- and manganese-dependent serine/threonine protein phosphatases [39]. To better characterize the specificity of GSK2830371 as a WIP1 antagonist, we performed *in vitro* reactions using phosphatases from both metal-independent (PP2A) and -dependent (PPM) subclasses of the type 2 protein phosphatase family. Specifically, we measured the activity of each phosphatase on a generic substrate, RRA(pT)VA, in the presence or absence of GSK2830371 (Fig. 3A). Our results indicate that of the phosphatases tested only WIP1 activity is significantly inhibited by GSK2830371 (96.5% inhibition relative to DMSO control) (Fig. 3A). We next extended these findings to confirm that GSK2830371 can block WIP1-mediated dephosphorylation of its physiological substrates. WIP1 acts on a number of substrates involved in the DNA damage response containing pS/pTQ motif including p53 (serine 15) and histone H2AX (serine 139) [13,40,41]. GSK2830371 inhibited WIP1 phosphatase activity on multiple WIP1 substrate phosphopeptides (S2 Fig.), including p53 (serine 15) and histone H2AX (serine 139) with IC_50_ values of 394 nM and 123 nM, respectively (Fig. 3B). These results, together with previous analyses of biochemical selectivity, indicate that GSK2830371 is a potent and specific antagonist of WIP1.

### *TP53* Wild-Type Neuroblastoma Cell Lines are Sensitive to WIP1 Inhibition

**Fig 4:**
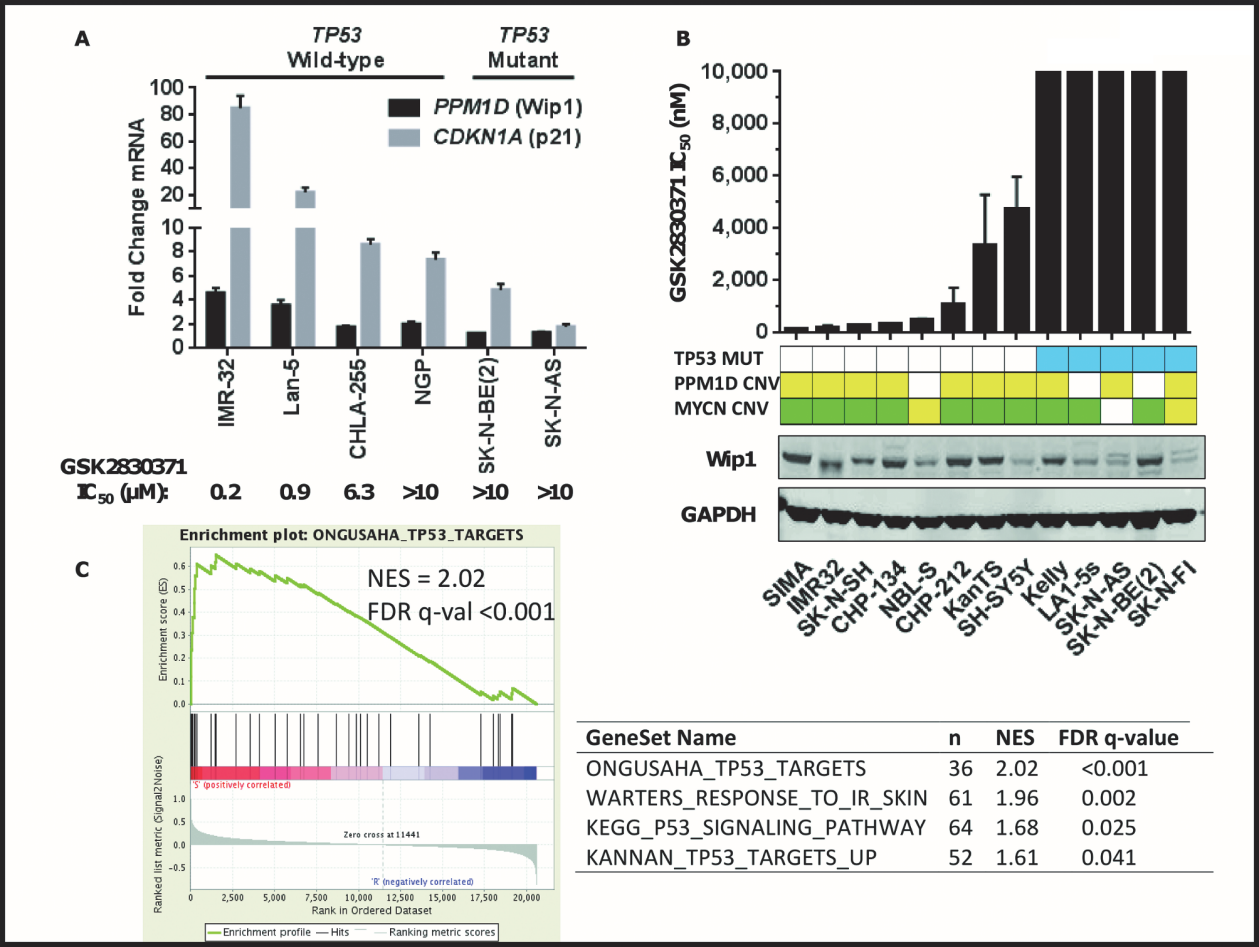
Neuroblastoma cell lines with wild-type *TP53* and functional p53 response are sensitive to GSK2830371. **A**, As a measure of p53 functional response, six neuroblastoma cell lines with different sensitivities to GSK2830371 (indicated by IC_50_ value in a proliferation assay) and *TP53* mutational status were exposed to ionizing radiation and allowed to recover for 1 hour, after which RT-PCR was carried out to measure changes in *PPM1D* and *P21* transcript levels. Data represent fold change relative to untreated cells (mean ± SD). **B**, Anti-proliferative effect of GSK2830371 in a seven day cell proliferation assay. Data represent mean ± SD. Copy number for *PPM1D* and *MYCN* is represented as green for gain of >6 copies; yellow for 2–6 copies. WIP1 protein level was determined by western blotting of whole cell lysates from a panel of neuroblastoma cell lines. **C**. GSEA enrichment plot showing a *TP53* gene signature that is significantly enriched in the sensitive group (S) relative to the resistant group (R) of neuroblastoma cell lines (see Table 1). Additional *TP53* genesets tested by GSEA and their respective numbers of genes (n), normalized enrichment scores (NES) and FDR q values are shown.

Due to the high frequency of WIP1 mRNA overexpression and amplification in neuroblastomas [22], we hypothesized that neuroblastoma cell lines might be sensitive to GSK2830371. To test this hypothesis, we analyzed p53 functional status and evaluated anti-proliferative activity in a small panel of neuroblastoma cell lines (Fig. 4A). Since we previously observed that lack of *TP53* mutation is an important determinant of WIP1 inhibitor sensitivity, we sequenced full length *TP53* in each cell line (Table 1; S1 Table). Our results indicate that the two *TP53* mutant cell lines, SK-N-BE(2) and SK-N-AS, are resistant to GSK2830371 while *TP53* wild-type cell lines have varying degrees of sensitivity ranging from 0.2 μM for IMR-32 to >10 μM for NGP cell line (Fig. 4A). To analyze the level of p53 pathway activity in these cell lines, we treated each with ionizing radiation and measured transcriptional activation of p53 target genes, *CDKN1A* (p21) and *PPM1D* itself (Fig. 4A). The cell line with greatest sensitivity to GSK2830371, IMR-32, had the most robust transcriptional response with 85- and 5-fold increases in p21 and WIP1, respectively, over non-irradiated samples. Lan-5 had intermediate response changes (22-fold for p21 and 3.5-fold for WIP1), while CHLA-255 and NGP had relatively weak responses (<10-fold for p21 and <2-fold for WIP1). Neither of the *TP53* mutant cell lines showed increased Wip1WIP1 mRNA levels, but SK-N-BE(2) did have a 5-fold increase in p21. These results suggest that sensitivity of neuroblastoma cell lines to growth inhibition by GSK2830371 is dictated not only by *TP53* mutational status, but also by the magnitude of a functional p53 response.

To expand upon these findings, we sequenced *TP53* full length and determined a relationship, if any, with GSK2830371 antiproliferative response in a larger panel of neuroblastoma cell lines. In this panel of 13 cell lines, once again *TP53* mutational status clearly determines sensitivity or resistance to GSK2830371. Seven of 11 *TP53* wild-type cell lines tested had some degree of sensitivity to GSK2830371 while all 5 *TP53* mutant cell lines were resistant (Fig. 4B; Table 1).

We performed GSEA analysis using publicly available gene expression microarray data for 12 of 16 cell lines that were differentially sensitive to GSK2830371 in our panel. By interrogating independently generated *TP53* gene sets, GSEA revealed a statistically significant (FDR q<0.05) enrichment (NES >1.5) in the sensitive group (n = 6) relative to the resistant group (n = 6) of lines (Fig. 4C).

Since WIP1 (*PPM1D*) is reported to be altered in neuroblastoma, we investigated if there was a relationship between the sensitivity of neuroblastoma cell lines and *PPM1D* copy number status. Among cell lines with a gain in *PPM1D* by copy number analysis (SIMA, IMR32, CHP-134, CHP-212, KANTS, SK-SY5Y, Kelly, SK-N-AS, SK-N-FI) majority of cell lines with wild-type *TP53* showed sensitivity to GSK2830371 (Fig. 4B). We further noted that in this set of cell lines, WIP1 protein levels did not correlate with sensitivity to GSK2830371A which could be explained by the lack of a correlation between WIP1 protein levels and copy number status.

*MYCN* is a commonly amplified gene in neuroblastoma tumors, therefore we determined its copy number levels as well in the same panel of cell lines [42]. We found that the large majority of these cell lines (Table 1) harbored a gain or amplification of *MYCN*, suggesting that it is not likely a determinant of sensitivity to WIP1 inhibition.

### GSK2830371 Inhibits WIP1 Cellular Signaling in a Wild-Type *TP53* Dependent Manner

**Fig 5:**
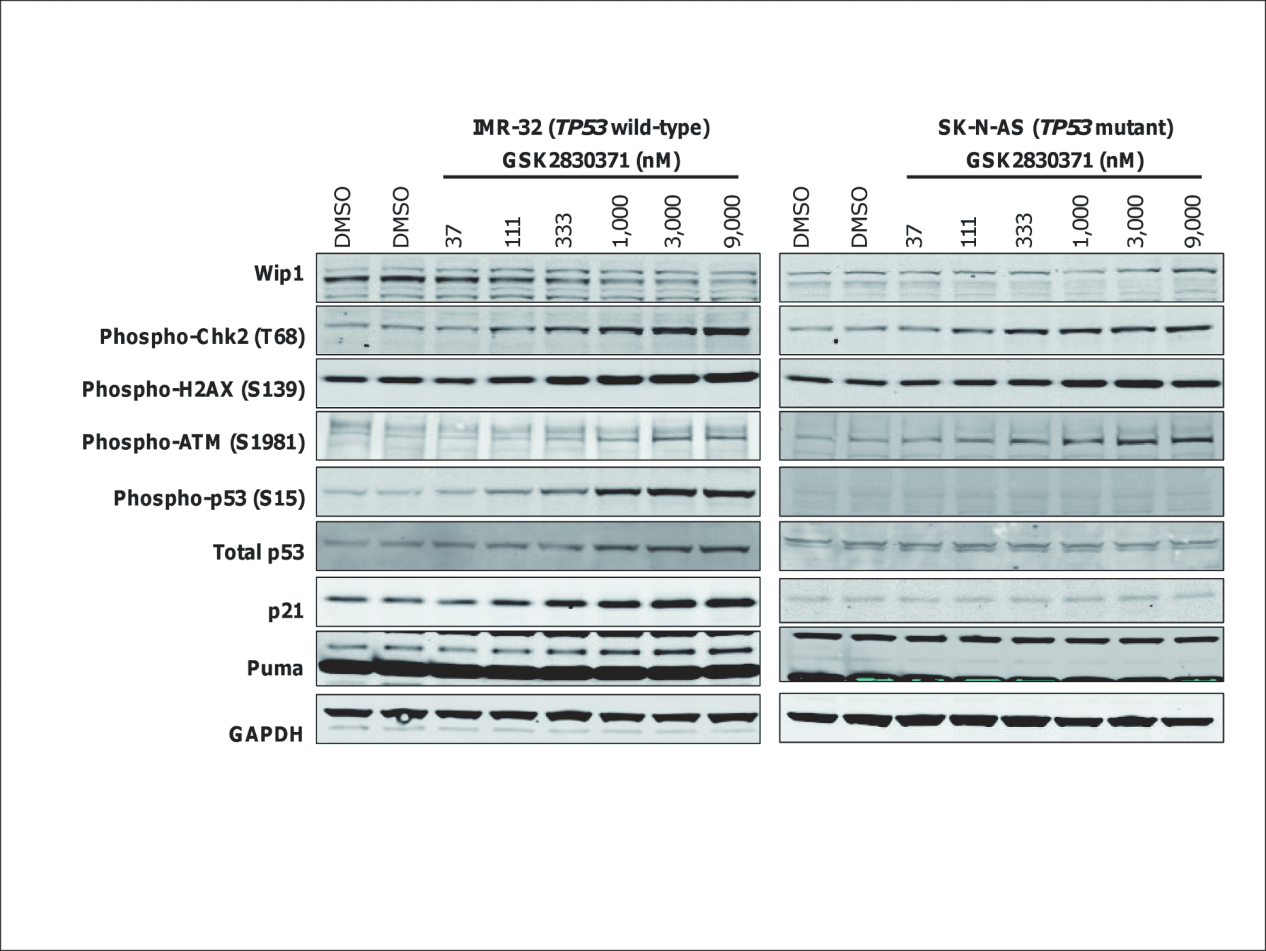
GSK2830371 inhibits WIP1 signaling differentially in *TP53* wild-type vs. mutant neuroblastoma cell lines. *TP53* wild-type (IMR-32) and mutant (SK-N-AS) cells were treated with indicated concentrations of GSK2830371 or DMSO for six hours. Cell lysates were analyzed by western blotting with antibodies to the indicated total and phospho-proteins. In both cell lines, GSK2830371 causes a concentration-dependent decrease in WIP1 protein and increases in WIP1 phospho-substrates, CHK2 (T68), H2AX (S139), and ATM (S1981). However, total and phospho-p53 (S15), and p53 transcriptional targets, p21 and PUMA, are only increased in the *TP53* wild-type cells.

WIP1 dephosphorylates several cellular substrates which could be relaying a survival signal to tumor cells, including ATM, checkpoint kinases, CHK1 and CHK2, H2AX, and p53. We wanted to understand which of these are critical to the antiproliferative response upon the inhibition of WIP1 in *TP53* wild-type neuroblastoma cell lines. We exposed the sensitive IMR-32 (*TP53* wild-type) cell line and the resistant SK-N-AS (mutant) cell line to increasing concentrations of GSK2830371 for six hours and examined known WIP1 substrates and p53 response proteins by western blot (Fig. 5). Both cell lines show increases in phospho-CHK2, -H2AX, and -ATM when treated with GSK2830371, whereas only IMR-32 shows increased phospho-p53 (S15) and total p53 as well as p53 transcriptional targets, p21 and PUMA (Fig. 5). GSK2830371 has the unique capability of destabilizing WIP1 itself, which we have shown to be due, in part, to proteasomal activity [33]. Despite the large differences in WIP1 basal protein level between IMR-32 and SK-N-AS, this destabilization occurs in both cell lines. These findings strongly suggest that neuroblastoma cell sensitivity to GSK2830371 is primarily dependent on p53 activity, and that effects on additional WIP1 substrates that lie upstream of p53 will contribute to growth inhibition only in the context of a functional P53 pathway. It should be noted that basal levels of other known phospho-substrates of WIP1, p38(T180) and CHK1(S345), were very low or undetectable and showed no increase upon treatment with GSK2830371 in these cell lines (data not shown).

### WIP1 Inhibition Augments the Antiproliferative Effects of Chemotherapeutic Agents

**Fig 6:**
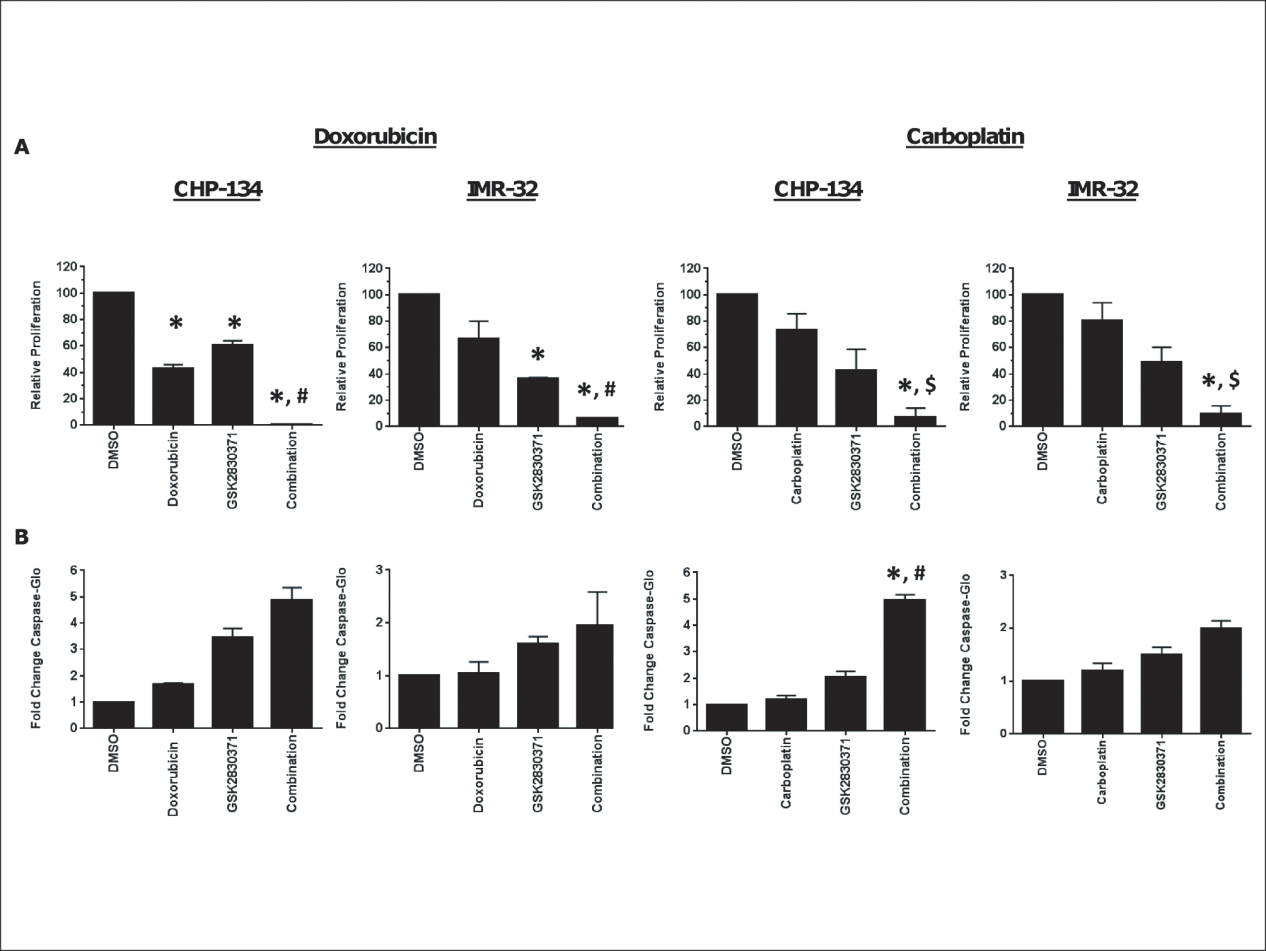
GSK2830371 has synergistic anti-proliferative activity with chemotherapeutic agents in neuroblastoma cells. CHP-134 and IMR-32 cells were exposed to 1 μM GSK2830371 for one hour prior to addition of doxorubicin (4 nM proliferation; 1 nM caspase) or carboplatin (1 μM proliferation; 10 μM caspase). CellTiter-Glo (A) was added to plates at 72 hours and caspase-Glo 3/7 (B) was added to separate duplicate plates at 24 or 48 hours post-drug addition. Data represent mean ± SD. *p<0.05 vs DMSO control, ^#^p<0.05 vs both single agents, ^$^p<0.05 vs doxorubicin or carboplatin alone, using t-test, (2 tailed, unequal variance).

Although GSK2830371 inhibits proliferation of neuroblastoma cell lines, we wondered whether WIP1 inhibition might exacerbate the antiproliferative effects of drugs that also induce apoptosis via p53. We exposed two wild-type *TP53* cell lines, CHP-134 and IMR-32, to GSK2830371 for one hour prior to treatment with doxorubicin or carboplatin, neuroblastoma standard of care induction drugs, for 3 days. Although GSK2830371 and each chemotherapeutic agent alone caused some degree of growth inhibition, the combination reduced the viability significantly more than either treatment alone (Fig. 6A). This effect was more pronounced in CHP-134 cells where combination of GSK2830371 with doxorubicin or carboplatin showed a synergistic response using mutually nonexclusive combination index analysis (CI = 0.56 and 0.60, respectively). Additionally, these drug combinations potentiated caspase 3/7 induction, indicating a more substantial apoptotic response (Fig. 6B).

## Discussion

Neuroblastoma is a neural crest derived tumor that requires repression of p53 for tumorigenesis [32]. This aggressive malignancy accounts for almost 15% of all childhood cancer deaths primarily due to drug resistant relapsed disease. However, In the majority of cases reduced P53 functionality is attributable to alterations in upstream regulators like MDM2 and P14ARF, rather than direct mutation of *TP53* [29,43,44]. Consequently, most relapsed tumors are likely to retain sensitivity to P53 pathway activators like MDM2 antagonists [45]. Thus there is significant interest in using P53 activating therapies to improve responses to, and limit toxicity associated with conventional chemotherapeutic regimens.

The infrequency of the *TP53* mutation as well as the high frequency of WIP1 overexpression in neuroblastoma suggests the potential for WIP1 inhibitors to have therapeutic benefit [46–48]. In the current study, we have demonstrated that GSK2830371, a highly selective small molecule antagonist of WIP1, has P53-dependent antiproliferative activity in neuroblastoma cell lines. Among tested cell lines, sensitivity to GSK2830371 correlated strongly with a functional *TP53* phenotype, defined by both absence of *TP53* mutation and by a transcriptomic signature of P53 functionality. In our GSEA analysis, the latter was based on enrichment of multiple genesets, including canonical P53 response genes such as FAS and CDKN1A; these genesets incorporated transcriptional responses in different contexts and as such, describe a functional *TP53* phenotype, independent of mutation status. Notably, 2 of the 4 wild-type *TP53* cell lines that were resistant to WIP1 inhibition were not enriched for the *TP53* functional genesets and may represent a diminished or defective *TP53* pathway. The growth inhibition of WIP1-inhibited neuroblastoma cells appears to be driven by hyperphosphorylation and stabilization of P53, resulting in an apoptotic response.

In addition to single agent activity, GSK2830371 also acts synergistically with neuroblastoma standard of care agents to reduce the viability of wild-type p53 neuroblastoma cells. Although, both CHP-134 and IMR-32 cell lines show synergistic anti-proliferative effect with GSK2830371 and doxorubicin or carboplatin, induction of caspase activity with combination was more pronounced in CHP-134 cell line. Both cell lines have functional p53 and are sensitive to GSK2830371, and thus the differences in the caspase induction with combination likely represents diversity of responses observed in different cell lines and patients to various anti-cancer agents which might be related to genetic diversity. Overall, our findings are in agreement with related studies that observed combination benefits in tumors between treatments that silenced or inhibited Wip1 activity and DNA damaging agents [22,49,50]. Therefore, it is possible that the synergy seen from combining GSK2830371 and doxorubicin or carboplatin is due to restored DNA damage response resulting from inhibition of WIP1. In summary, our results provide evidence that a selective Wip1 inhibitor causes targeted antiproliferative effects on *TP53* wild-type neuroblastoma with potential additive benefit when combined with DNA damaging chemotherapies, currently used to treat patients with this disease.

## Supporting Information

S1 Fig WIP1 expression in normal tissues. Survey of *PPM1D* (WIP1; 204566_at) mRNA expression from >25K microarray expression profiles across multiple human tissues. Overexpression is determined above the indicated threshold (see Materials and Methods).
(TIF)

S2 Fig GSK2830371 inhibits the magnesium-dependent phosphatase activity of WIP1. Free phosphate is released from four WIP1 substrate peptides in the complete reaction (Enz, Peptide, Mg). GSK2830371 inhibits this phosphate release to levels similar to that of the “no enzyme” and “no Mg” conditions.
(TIF)

S1 Table Primers used for sequencing of *TP53* genomic DNA. (DOCX)
