# Supplementary figures and images for: WIP1 Phosphatase as a Potential Therapeutic Target in Neuroblastoma
Source: PLoS One. 2015 Feb 6;10(2):e0115635. doi: 10.1371/journal.pone.0115635 (PMC4319922; doi:10.1371/journal.pone.0115635)

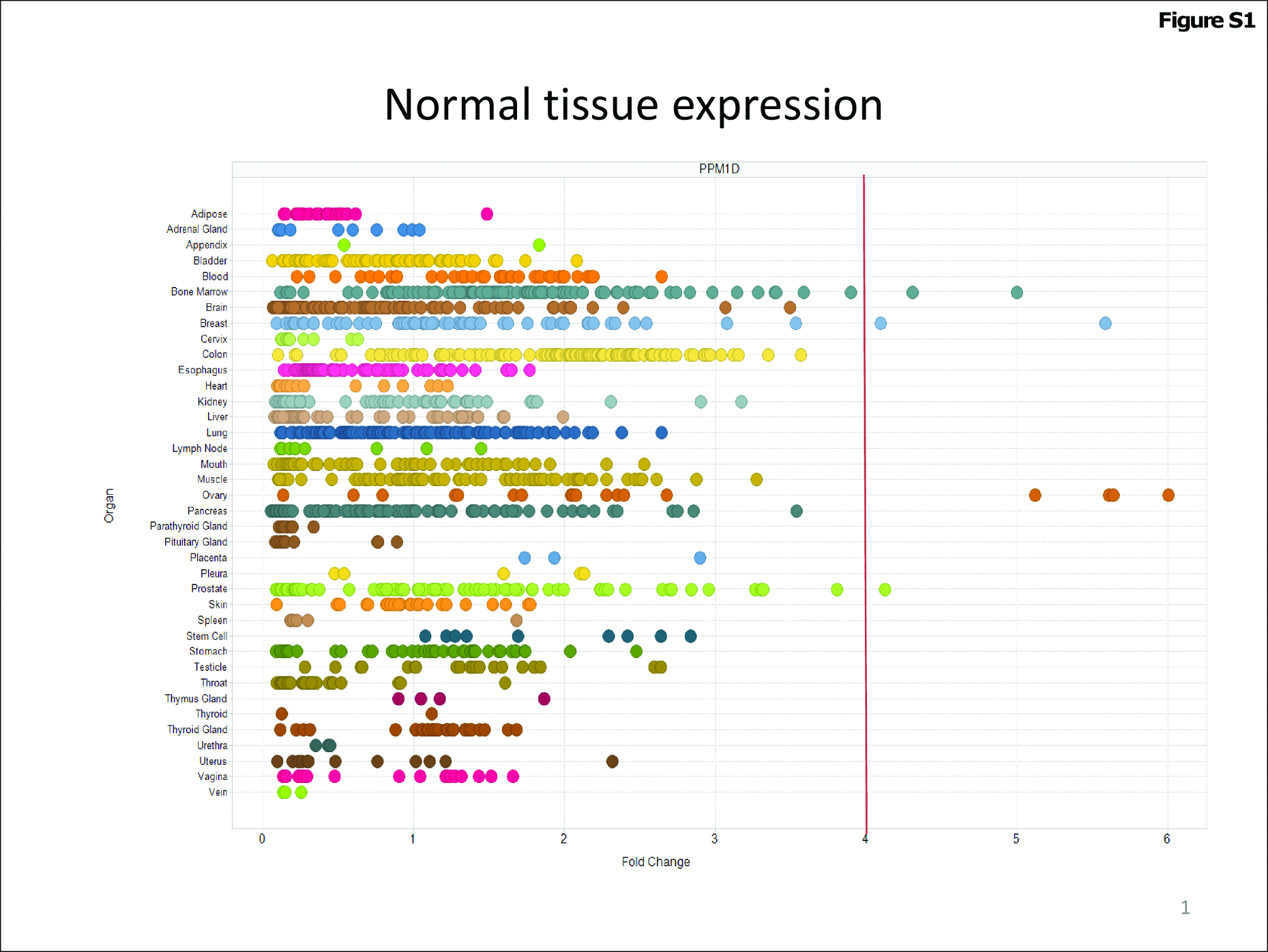

Supplement: S1 Fig — Survey of PPM1D (WIP1; 204566_at) mRNA expression from >25K microarray expression profiles across multiple human tissues. Overexpression is determined above the indicated threshold (see Materials and Methods). (TIF) [file pone.0115635.s001.tif]

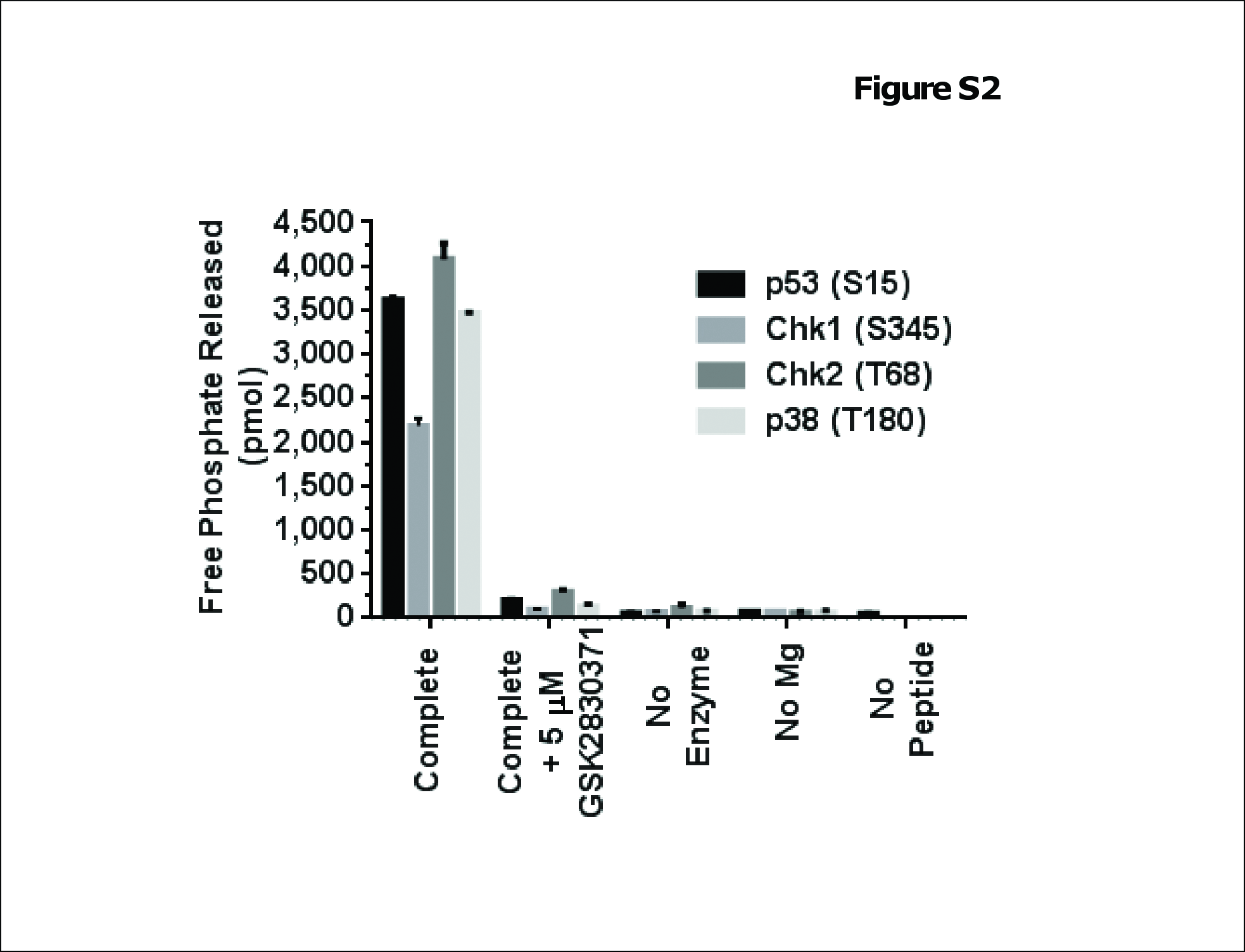

Supplement: S2 Fig — Free phosphate is released from four WIP1 substrate peptides in the complete reaction (Enz, Peptide, Mg). GSK2830371 inhibits this phosphate release to levels similar to that of the “no enzyme” and “no Mg” conditions. (TIF) [file pone.0115635.s002.tif]
