# Supplementary material for: WIP1 Phosphatase as a Potential Therapeutic Target in Neuroblastoma
Source: PLoS One. 2015 Feb 6;10(2):e0115635. doi: 10.1371/journal.pone.0115635 (PMC4319922; doi:10.1371/journal.pone.0115635)
Supplement: S1 Table — (DOCX) [file pone.0115635.s003.docx]

**Table S1: Primers used for sequencing of *TP53* genomic DNA**

| **Region** | **Primer sequence (5’ 🡪 3’; F: forward; R: reverse)** |
| --- | --- |
| *TP53*.Exon2-3 | F:CTCATGCTGGATCCCCAC |
| *TP53*.Exon4 | F:CGTTCTGGTAAGGACAAGGG |
| *TP53*.Exon5-6 | F:CTTTATCTGTTCACTTGTGCCC |
| *TP53*.Exon7 | F:CTGCTTGCCACAGGTCTCC |
| *TP53*.Exon8-9 | F:GGGACAGGTAGGACCTGATTTC |
| *TP53*.Exon10 | F:cttgaaccatcttttaactcagg |
| *TP53*.Exon11 | F:GGAAAAGGGGCACAGACC |
| *TP53*.Exon2-3 | R:TGGGTGAAAAGAGCAGTCAG |
| *TP53*.Exon4 | R:GGAATCCCAAAGTTCCAAAC |
| *TP53*.Exon5-6 | R:CAGCAGGAGAAAG |
| *TP53*.Exon7 | R:TGATGAGAGGTGGATGGGTAG |
| *TP53*.Exon8-9 | R:GGCATTTTGAGTGTTAGACTGG |
| *TP53*.Exon10 | R:GGAATCCTATGGCTTTCCAAC |
| *TP53*.Exon11 | R:GCAGGGGAGGGAGAGATG |
